# Comparative evaluation of chlorhexidine, er, cr: ysgg laser, and iron oxide nanoparticles as dentin disinfectants: effects on streptococcus mutans inhibition and glass ionomer bond strength in primary molars

**DOI:** 10.12669/pjms.42.4.15044

**Published:** 2026-04

**Authors:** Faisal Ali bin Abbooud AlQhtani, Zuhair Motlak Alkahtani, Ahmed Ali M Albariqi, Muhammad Abdullah Kamran

**Affiliations:** 1 Faisal Ali bin Abbooud AlQhtani Consultant in Pediatric Dentistry, Department of Pediatric Dentistry and Orthodontic Sciences, College of Dentistry, King Khalid University. Abha, KSA; 2 Zuhair Motlak Alkahtani Department of Pediatric Dentistry and Orthodontics, College of Dentistry, King Khalid University. Abha, KSA; 3 Ahmed Ali M Albariqi Department of Periodontics and Community Dentistry, College of Dentistry, King Khalid University. Abha, KSA; 4 Muhammad Abdullah Kamran Department of Pediatric Dentistry and Orthodontic Sciences, College of Dentistry, King Khalid University. Abha, KSA

**Keywords:** Bond strength, Cavity disinfection, Chlorhexidine, Er, Cr: YSGG laser, Iron oxide nanoparticles, Glass ionomer cement, Primary teeth, Streptococcus mutans

## Abstract

**Objective:**

To find out the influence of different dentin disinfectants—chlorhexidine (CHX), Er, Cr: YSGG laser (ECYL), and iron oxide nanoparticles (Fe_3_O_4_NPs)—on inhibition of *Streptococcus mutans* and shear bond strength (SBS) of glass ionomer cement (GIC) in primary molars.

**Methodology:**

This in vitro study was conducted at King Khalid University, approved under IRB# KKU 2025-2026-161. The study duration was three months, 15^th^ September, 2025 - 20^th^ December, 2025. Forty extracted primary second molars with ICDAS scores of five were selected. CAD was identified through visual inspection, surface hardness assessment, and dye staining. Teeth were randomly divided into four groups (n=10): Group-I (no disinfection), Group-II (2% CHX), Group-III (ECYL at 2.5W, 20Hz), and Group-IV (Fe_3_O_4_NPs). Antibacterial efficacy against *S. mutans* was assessed using the agar diffusion test. GIC restorations were placed and subjected to thermocycling. SBS testing was performed using a universal testing machine, and failure modes were analyzed under stereomicroscopy. Data were analyzed using one-way ANOVA and Tukey’s post hoc test (α=0.05).

**Results:**

CHX (Group-II) demonstrated the largest zone of inhibition (15.95±1.54 mm). The control group showed the smallest inhibition zone (6.24±1.13 mm). ECYL (Group-III) exhibited the highest SBS (7.33±0.34 MPa), statistically superior to all groups (p<0.05). Fe_3_O_4_NPs (Group-IV) displayed the lowest bond strength (4.02±0.25 MPa). Mixed failure patterns predominated across all groups.

**Conclusion:**

Er, Cr: YSGG laser demonstrated optimal performance as a cavity disinfectant, combining effective antimicrobial activity with superior bond strength preservation in primary molars.

## INTRODUCTION

Dental caries in primary dentition remains a significant public health challenge, with *Streptococcus mutans (S.mutans*) identified as the principal etiological agent.^1^ Preservation of primary teeth is critical for proper growth, development, and space maintenance in pediatric patients.^2^ Glass ionomer cement (GIC) has emerged as the material of choice for primary tooth restoration due to its chemical bonding to tooth structure, sustained fluoride release, biocompatibility, and coefficient of thermal expansion similar to natural dentition.^1^

Minimally invasive dentistry advocates selective removal of infected dentin while preserving carious-affected dentin (CAD), thereby maximizing residual tooth structure.^3^ However, CAD presents significant challenges for adhesive restorations. The microstructural alterations in CAD—including dentinal tubule occlusion by acid-resistant mineral crystals and reduced mineral density—result in substantially diminished shear bond strength (SBS) compared to sound dentin.^4^

Additionally, residual bacteria within CAD can precipitate secondary caries and restoration failure.^5^ These limitations necessitate effective cavity disinfection protocols before restoration placement. Chlorhexidine (CHX), a cationic biguanide antimicrobial agent, has demonstrated broad-spectrum antibacterial efficacy and matrix metalloproteinase (MMP) inhibition properties, theoretically enhancing dentin-restoration interface longevity.^6^ While Nisar et al. reported that CHX application does not significantly improve composite-to-CAD bond strength in primary molars, its effects on GIC adhesion to primary tooth CAD remain inadequately characterized.^2^

Er, Cr: YSGG laser (ECYL), operating at 2,780 nm wavelength, offers an alternative disinfection modality through hydrokinetic ablation that simultaneously eliminates bacteria and smear layer while creating micro-retentive surface patterns.^7^,^8^ However, conflicting data exist regarding laser-mediated bond strength outcomes, with variable results attributed to parameter inconsistencies across studies.^9^,^10^ Standardized protocols for primary molar CAD treatment are lacking. Recent advances in nanotechnology have introduced iron oxide nanoparticles (Fe_3_O_4_NPs) as promising antimicrobial agents. FDA-approved for biomedical applications, it includes MRI contrast enhancement. Fe_3_O_4_NPs demonstrate potent antibacterial and antibiofilm properties through reactive oxygen species generation.^11^ Preliminary investigations suggest Fe_3_O_4_NPs incorporation into dental adhesives maintains or enhances bond strength to tooth structure.^12^ However, their efficacy against *S. mutans* and influence on GIC-to-CAD bond integrity when employed as cavity sterilants remain unexplored.

The present study tests the null hypotheses that (1) no significant differences exist in antimicrobial efficacy against *S. mutans* among CHX, ECYL, Fe_3_O_4_NPs, and untreated controls, and (2) no significant differences exist in GIC-to-CAD bond strength among cavity disinfection protocols. The objective is to comprehensively evaluate three distinct disinfection modalities (CHX, ECYL, Fe_3_O_4_NPs) through dual-outcome assessment of antimicrobial efficacy and mechanical bond performance in CAD primary molars.

## METHODOLOGY

This in-vitro study adhered to the Checklist for Reporting In-vitro Studies CRIS guidelines. Forty non-restored primary second molars were collected. Duration of the study was three months, from 15^th^ September 2025 to 20^th^ December 2025.

### Inclusion criteria:

It required specimens exhibiting ICDAS (International Caries Detection and Assessment System) scores of five, indicating moderate to extensive cavitated lesions with visible dentin involvement. Specimens with fractures, cracks, non-carious lesions, or previous restorations were excluded. Specimens were stored in 0.5% chloramine-T solution at room temperature until experimental procedures.^13^

### Ethical Approval:

After taking informed parental consent institutional ethical approval was obtained Ref. # IRB# KKU 2025-2026-161.

### CAD Procurement and Standardization:

CAD identification used three criteria: visual-tactile examination with a sharp dental explorer to assess surface hardness, application of a caries detector dye (Kuraray Co., Ltd., Osaka, Japan), and stereomicroscopic verification. Infected dentin exhibiting dark coloration was carefully excavated using sterile low-speed round burs, preserving the light pink CAD layer.^5^ To create standardized bonding surfaces, occlusal thirds were sectioned perpendicular to the long axis using a diamond disk under copious water irrigation. Exposed dentin surfaces were sequentially polished with 400-grit and 600-grit silicon carbide papers (Bosch C355, Switzerland) under cooling water to establish a uniform smear layer and eliminate surface irregularities. Specimens were then embedded in auto-polymerizing acrylic resin (Probase, Ivoclar Vivadent, Spain) within cylindrical molds, leaving the prepared CAD surface exposed.^14^

### Experimental Groups and Disinfection Protocols:

Specimens were randomly allocated into four groups (n=10) using computer-generated randomization:

### Group-I (Control):

No disinfection applied; CAD surfaces rinsed with distilled water for 10 seconds and air-dried.

### Group-II (Chlorhexidine):

2% CHX digluconate solution (Consepsis, Ultradent Products Inc., South Jordan, UT, USA) applied with sterile microbrush for 20 seconds, followed by 10 seconds water rinse and 5-second air-drying.^8^

### Group-III (Er, Cr: YSGG Laser):

ECYL (Waterlase, Biolase, California, USA) with MZ8 sapphire tip positioned 2 mm from CAD surface, operated at standardized parameters: 2,780 nm wavelength, 0.5 W power, 30 Hz frequency, 60 seconds exposure time, with air-water spray ratio of 65%:55%. Post-irradiation surfaces were rinsed for 10 seconds and gently air-dried.^15^

### Group-IV (Fe_3_O_4_NPs Nanoparticles):

**Fig.1:**
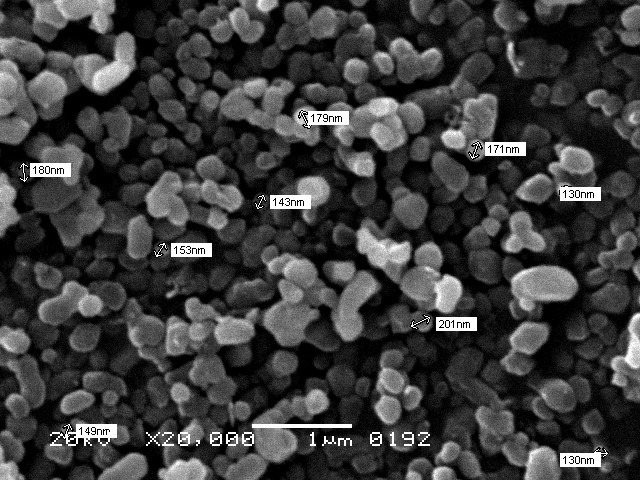
SEM of Fe_3_O_4_NPs

Fe_3_O_4_NPs (25-50 nm diameter; US Research Nanomaterials Inc., Houston, USA) prepared as 1% w/v aqueous suspension (10 g powder in 1 L deionized water), ultrasonically agitated for 30 minutes to ensure homogeneous dispersion. Suspension applied to CAD surfaces for 60 seconds using a sterile microbrush, followed by a 10 seconds water rinse and air-drying.^16^ (Fig.1)

### Antimicrobial Efficacy Assessment:

*Streptococcus mutans* ATCC 25175 was cultured on blood agar medium (supplemented with 5% sheep blood) and incubated anaerobically (5-10% CO_2_) at 37°C for 48 hours. Bacterial inoculum was standardized to 0.5 McFarland turbidity (1.5 × 10^8^ CFU/mL). Agar well diffusion assays were performed using Mueller-Hinton agar plates, autoclaved at 121°C for 20 minutes. Standardized bacterial suspension (0.1 mL) was evenly spread using sterile swabs. Four equidistant wells (8 mm diameter) were created per plate using a sterile cork borer. Each well received 100 μL of the respective test agent, with three replicates per group. Plates were incubated at 37°C for 48 hours under anaerobic conditions. Inhibition zones were measured in millimeters using digital calipers (accurate to 0.01 mm) by two calibrated examiners blinded to group allocation, with measurements taken in perpendicular directions and averaged.^17^

### Glass Ionomer Cement Restoration:

CAD surfaces were conditioned with 25% polyacrylic acid (Ketac Conditioner, 3M ESPE, Seefeld, Germany) for 10 seconds, water-rinsed for 10 seconds, and gently air-dried for five seconds to maintain moist dentin. Conventional GIC (Ketac Molar Easymix, 3M ESPE) was hand-mixed according to manufacturer specifications (1:1 powder-to-liquid ratio for 30 seconds). Standardized GIC cylinders (5 mm diameter × 3 mm height) were built using Tygon tubing matrices positioned perpendicular to CAD surfaces. Following a five-minute initial setting, cylinders were removed, and specimens were stored at 100% humidity at 37°C for 24 hours to complete cement maturation.^18^

### Thermocycling Protocol:

All specimens underwent artificial aging via thermocycling (SD Mechatronik GMBH, Feldkirchen-Westerham, Germany) for 10,000 cycles between 5°C and 55°C water baths (simulating approximately one year of clinical service), with 30-second dwell time and 5-second transfer interval.^19^

### SBS Testing:

Specimens were mounted in a universal testing machine (Laryee Technology Co., Ltd., Beijing, China) with shear load applied parallel to the GIC-dentin interface at 0.5 mm/min crosshead speed until failure. Maximum load at failure (N) was recorded, and SBS was calculated:

**SBS (MPa) = Load (N) / Bonding area (mm²)**.

### Failure Mode Analysis:

Fractured interfaces were examined under stereomicroscopy (AZ100M, Nikon, Tokyo, Japan) at 40× magnification and classified as:

adhesive (interfacial failure),

cohesive (within GIC or dentin), or

mixed (combination pattern).

### Statistical analysis:

Data were analyzed using SPSS version 26.0 (IBM Corp., Armonk, NY). Normality was assessed using the Shapiro-Wilk test. One-way ANOVA with Tukey HSD post-hoc tests evaluated intergroup differences. Statistical significance was set at α=0.05

## RESULTS

### Inhibition zone against S.Mutans:

Table-I displays the inhibition zone of *S. mutans* following different cavity disinfectants. The widest zone of inhibition was presented by Group-II (CHX) (15.95±1.54 mm). Whereas the narrowest zone was displayed by Group-I (No disinfection) (6.24±1.13 mm). Intergroup comparison analysis showed that Group-II, Group-III (ECYL) (15.43±1.43 mm), and Group-IV (Fe_3_O_4_NPs) (15.77±1.33 mm) demonstrated no significant difference in inhibition zone thickness (p>0.05)

**Table-I:** The mean± standard deviation values of the inhibition zone of Streptococcus mutans following different cavity disinfectants.

Tested groups	Inhibition zone Mean ± SD (mm)	p-value!
Group-I: No disinfection	6.24±1.13 ^a^	< 0.05
Group-II: CHX	15.95±1.54 ^b^	
Group-III: ECYL	15.43±1.43 ^b^	
Group-IV: Fe_3_O_4_NPs	15.77±1.33 ^b^	

!ANOVA Chlorhexidine (CHX), Er, Cr: YSGG laser (ECYL), Iron oxide nanoparticles (Fe_3_O_4_NPs) The different superscript denotes a statistically significant difference (p<0.05)

### SBS analysis:

Table-II demonstrated the SBS of conventional GIC to CAD after applying various cavity sterilants. Group-III ECYL (7.33±0.34 MPa) presented the highest bond SBS. Whereas Group-IV (Fe_3_O_4_NPs) (4.02±0.25 MPa) exhibited the lowest bond strength. Intergroup comparative analysis discovered that Group-I (Control) (4.21±0.1 MPa), Group-II (CHX) (4.55±0.23 MPa), and Group-IV demonstrated comparable bond strength outcomes (p>0.05)

**Table-II:** SBS of conventional GIC material to CAD after applying various cavity sterilants.

Tested groups	SBS Mean ± SD (MPa)	p-value!
Group-I: No disinfection	4.21±0.19 ^b^	< 0.05
Group-II: CHX	4.55±0.23 ^b^	
Group-III: ECYL	7.33±0.34 ^a^	
Group-IV: Fe_3_O_4_NPs	4.02±0.25 ^b^	

!ANOVA Chlorhexidine (CHX), Er, Cr: YSGG laser (ECYL), Iron oxide nanoparticles (Fe_3_O_4_NPs) The different superscript denotes a statistically significant difference (p<0.05)

### Failure Mode Analysis:

**Fig.2:**
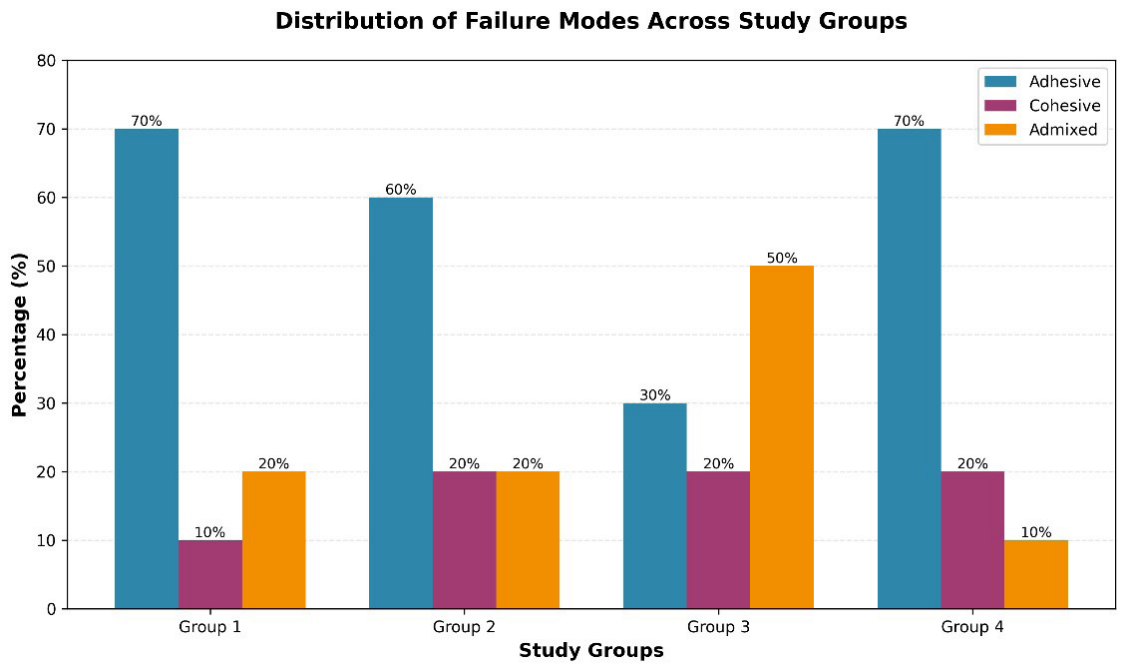
Distribution of failure modes in percentages across various study groups.

Adhesive failures (interfacial debonding at the GIC-dentin junction) predominated in Groups I, II, and IV (70%, 60%, and 70%, respectively), indicating weak bonding at the restoration-tooth interface. In contrast, Group-III (Er, Cr: YSGG laser) exhibited primarily mixed failures (50%) and the lowest adhesive failure rate (30%), suggesting superior interfacial bonding where the fracture locus shifted from the interface into the bulk materials (Fig.2).

## DISCUSSION

This study was predicated on dual null hypotheses: first, that cavity disinfectants would demonstrate no significant difference in antibacterial efficacy compared to the control; second, that no significant variation would exist in the SBS of GIC to CAD following cavity disinfectant pretreatment. The experimental findings comprehensively rejected the first hypothesis, as all tested groups exhibited significantly superior antimicrobial activity against Streptococcus *mutans* compared to the control. The second hypothesis was partially rejected, since only Er, Cr: YSGG laser-treated specimens demonstrated significantly enhanced bond strength relative to the control group.

The antimicrobial results revealed that all experimental groups displayed markedly superior antibacterial efficacy against *S. mutans* compared to the control. Fe_3_O_4_NPs demonstrate considerable promise for dental caries and root canal disinfection, attributable to their potent antibacterial and anti-biofilm properties.^20^ The principal antibacterial mechanism of Fe_3_O_4_NPs involves reactive oxygen species (ROS) generation, which subsequently elevates lipid peroxidation levels, diminishes antioxidant enzyme activity, and induces protein aggregation.^11^ These nanoparticles additionally provoke intracellular iron overload, culminating in severe cellular damage and eventual bacterial death.^11^ Ghorbanizadeh and colleagues reported that Fe_3_O_4_NPs exhibited more pronounced antibacterial effects than CHX, corroborating the present findings.^20^

The antimicrobial efficacy of Er, Cr: YSGG laser irradiation against *S. mutans* proved comparable to CHX, consistent with previous observations by Türkün and associates.^21^ The mechanical and thermal effects of laser energy facilitate the physical elimination of bacterial populations. This phenomenon occurs through significant absorption of laser energy by intracellular water molecules, which rapidly converts to heat, elevating local temperatures to bactericidal levels.^21^ The resultant thermal effects denature essential bacterial proteins and enzymes, ultimately causing cellular death.^22^ Conversely, CHX exerts its antimicrobial action through electrostatic interaction between its positive charge and the negatively charged bacterial cell wall, compromising membrane integrity, inducing cytoplasmic leakage of vital constituents including potassium ions, and ultimately causing bacterial death.^6^ This disruption manifests as bacteriostatic effects at lower concentrations or bactericidal activity at higher concentrations.^23^

Regarding bond strength outcomes, Er, Cr: YSGG laser-treated primary dentin surfaces exhibited superior GIC adhesion compared to other experimental groups, corroborating findings by Nagarathna Chikkanarasaiah and associates.^24^ The favorable performance of laser pretreatment stems from its ablation and micro-abrasion mechanisms, which create surface roughness analogous to acid-etched patterns. This enhanced topography increases the available surface area for adhesive bonding.^24^ CHX pretreatment demonstrated bond strength values comparable to the control group, aligning with observations by Yetkiner and coworkers, who reported that CHX-based cavity disinfectants did not compromise GIC bond strength.^25^

Additionally, previous investigations confirmed that 2% CHX solution does not adversely affect GIC marginal adaptation to dentin walls. Since GICs adhere to dental structures primarily through ionic exchange with hydroxyapatite, CHX application does not impair adhesion to either sound or caries-affected dentin.^4^ However, contradictory evidence suggests that CHX may interfere with resin-dentin bonding.^9^,^15^ Given the limited data regarding CHX effects on GIC-CAD bond integrity, further investigation remains warranted.

Concerning Fe_3_O_4_NPs, recent evidence indicates their incorporation into composite resins yields either beneficial or neutral effects on bond strength, depending on nanoparticle concentration and surface modification.^11^ However, the present study employed these nanoparticles as cavity disinfectants, revealing diminished bond strength compared to the control. We hypothesize that hydroxyapatite precipitation in the presence of iron results in calcium-deficient apatite formation, as iron occupies calcium-binding sites, a phenomenon previously described by Sutter and colleagues. This substitution may create a barrier that impairs GIC-dentin adhesion, thereby reducing bond strength.Er, Cr: YSGG laser-treated specimens predominantly exhibited admixed fractures, while the remaining groups displayed primarily adhesive failures, thereby confirming the measured bond strength values.

The study’s key strength lies in its clinically relevant multi-group comparative design evaluating three distinct cavity disinfectants (CHX, Er, Cr: YSGG laser, and iron oxide nanoparticles) simultaneously on both antimicrobial efficacy and GIC bond strength to caries-affected dentin in primary molars, with standardized CRIS-compliant methodology including thermocycling aging and blinded failure mode analysis.

### Study Limitations and Future Directions:

This investigation’s limitations stem from challenges in replicating oral environmental conditions, including thermal fluctuations, pH variations, sustained salivary exposure, and enzymatic activity. The in vitro framework, standardized methodology, and limited sample size preclude direct extrapolation to complex clinical scenarios until corroborated by additional in vitro studies employing more physiologically relevant conditions or through in vivo investigations.

### Clinical Innovation and Applications:

Er, Cr: YSGG laser pretreatment emerges as a dual-purpose intervention, providing effective cavity disinfection while simultaneously enhancing GIC adhesion to caries-affected primary dentin. This dual functionality potentially streamlines pediatric restorative protocols by eliminating the need for separate disinfection and surface conditioning steps.

## CONCLUSION

Er, Cr: YSGG laser demonstrated superior performance as a cavity disinfectant for carious-affected dentin in primary molars, achieving both potent antimicrobial efficacy against Streptococcus mutans and significantly enhanced glass ionomer cement bond strength. This dual benefit positions laser treatment as the optimal protocol for balancing infection control with restoration longevity in minimally invasive pediatric dentistry
